# Supplementary material for: Balance dynamics are related to age and levels of expertise. Application in young and adult tennis players
Source: PLoS One. 2021 Apr 15;16(4):e0249941. doi: 10.1371/journal.pone.0249941 (PMC8049250; doi:10.1371/journal.pone.0249941)
Supplement: Attachment — Submitted filename: Response to Reviewers.docx [file pone.0249941.s003.docx]

Dear editor and reviewers,

The authors would like to thank you for your advice and recommendations. As you will see, we have performed a revision of this manuscript based on your suggestions.

**Reviewers' comments:**

**Reviewer #3:**

**I. General Comments:**

**The purpose of this study was to assess the relationship between balance and tennis performance. Specifically, the purposes were: 1) to compare the balance of tennis players of different ages and levels of expertise, and 2) to analyze the relationship between balance and tennis performance (serving speed and accuracy). This subject is relevant to the fields of motor behavior. The modifications improved the quality of the manuscript. However, there are still some issues which warrant clarification. Specific comments are displayed below.**

Thank you very much for your recommendations. All the changes made are in red in the manuscript and each reviewer’s comments has been responded on a point-by-point basis:

**II. Specific Comments:**

1. **Regarding the concept of “balance”, the authors said that “The term balance has been clarified in the introduction section” (Response to Reviewers file). However, there are no differences between the revised Introduction and the previous one in respect to this issue. The authors just replaced “Balance ability” (line 48 – previous version) by “Balance” (line 41 – present version).**

Thank you for your comment. In the revised version, a definition was added at the beginning of the introduction to clarify the concept of balance control used in the manuscript. The authors have now rephrased the beginning of the introduction section to address more clearly this suggestion.

Lines 41-47: “*Balance is one of the main features of motor control as every human movement involves it. Balance has been defined as the ability of the neuromuscular system to integrate visual, vestibular and somatosensory feedback to control the body’s center of gravity projection (1, 2). Therefore, balance control has been frequently analyzed by the use of tasks that challenge the individual in maintaining the position of the body’s center of gravity vertically over the base of support (1, 2), and it has been commonly assessed through the body’s postural sway (3)”.*

In addition, in the *Balance task protocol section*, all the details about how the balance task was conducted are explained (Lines 139 – 148).

2. **In this reviewed manuscript the authors mentioned the number of trials performed in the balance protocol. According to authors “Participants performed one trial keeping this position for 60 seconds” (lines 145-146). Is one trial enough to provide reliable measurements of CoP? It’s necessary to justify and include the proper references.**

Thank you very much for your comment. Some studies have already proved that just 1 trial, even shorter than 60 s, is reliable to measure the COP (Caballero, Barbado & Moreno, 2015). The authors have chosen 60 seconds of measuring because several studies have used this time length in the literature (Caballero et al., 2016; Chapman et al., 2008; Lin, Seol, Nussbaum & Madigan, 2008). The references of the papers have been included in the method section. (Lines 146-148).

- Caballero, C., Barbado, D., & Moreno, F. J. (2015). What COP and Kinematic Parameters Better Characterize Postural Control in Standing Balance Tasks? Journal of Motor Behavior, 47(6), 550-562.
- Chapman DW, Needham KJ, Allison GT, Lay B, Edwards DJ. Effects of experience in a dynamic environment on postural control. Br J Sports Med. 2008;42(1):16-21.
- Lin, D., Seol, H., Nussbaum, M. A., & Madigan, M. L. (2008). Reliability of COP-based postural sway measures and age-related differences. Gait & Posture, 28(2), 337-342.
- Santos, B. R., Delisle, A., Lariviere, C., Plamondon, A., & Imbeau, D. (2008). Reliability of centre of pressure summary measures of postural steadiness in healthy young adults. Gait & Posture, 27(3), 408-415.

3. **Results revealed no Interaction (Age x Expertise) for service accuracy. So, the sentence “…multiple pair comparisons showed that older expert players (+18) significantly displayed better performance in their serve accuracy (MRE) than the youngest expert players (U12), while recreational players did not show significant differences according to age ranges” (lines 225-228) is still statistically unnecessary. If there was no interaction, the multiple pair comparisons do not matter.**

Thank you very much for your comment. The sentence have been removed from the results section.
